# Cross‐cohort change in adolescent outcomes for children with mental health problems

**DOI:** 10.1111/jcpp.13029

**Published:** 2019-04-15

**Authors:** Ruth Sellers, Naomi Warne, Andrew Pickles, Barbara Maughan, Anita Thapar, Stephan Collishaw

**Affiliations:** ^1^ Rudd Centre for Adoption Research and Practice School of Psychology University of Sussex Brighton UK; ^2^ Division of Psychological Medicine and Clinical Neurosciences MRC Centre for Neuropsychiatric Genetics and Genomics, School of Medicine Cardiff University Cardiff UK; ^3^ Department of Biostatistics and Health Informatics Institute of Psychiatry, Psychology and Neuroscience King's College London London, UK; ^4^ Social, Genetic and Developmental Psychiatry Centre Institute of Psychiatry, Psychology and Neuroscience King's College London London UK

**Keywords:** Child mental health, secular change, National Child Development Study, Avon Longitudinal Study of Parents and Children, Millennium Cohort Study

## Abstract

**Background:**

Child mental health problems are common. Previous studies have examined secular changes in their prevalence but have not assessed whether later outcomes have changed. We therefore aimed to test whether outcomes of child mental health problems have changed over a 40‐year period.

**Methods:**

Three cohorts were utilized: The National Child Development Study (NCDS:* N* = 14,544, aged 7 in 1965), the Avon Longitudinal Study of Parents and Children (ALSPAC:* N* = 8,188, aged 7 in 1998), and the Millennium Cohort Study (MCS:* N* = 13,192, aged 7 in 2008). Mental health problems at age 7 were identified using the parent‐reported Rutter‐A scale (NCDS) and Strengths and Difficulties Questionnaire (ALSPAC and MCS). Associated outcomes were compared across cohorts: age 11 social functioning, age 16 exam attainment and age 16 mental health.

**Results:**

Child mental health problems were common in each cohort (boys: 7.0%–9.7%; girls: 5.4%–8.4%). Child mental health problems became more strongly associated with social functioning problems (boys: NCDS OR = 1.95 (1.50, 2.53), MCS OR = 3.77 (2.89, 4.92); interaction *p* < .001; girls: NCDS OR = 1.69 (1.22, 2.33), MCS OR = 3.99 (3.04, 5.25), interaction *p* < .001), lower academic attainment for boys (NCDS OR = 0.49 (0.31, 0.78), ALSPAC OR = 0.30 (0.22, 0.41), interaction *p* = .009), and age 16 mental health problems (boys: NCDS *d*′* *= 0.55 (0.38, 0.72), ALSPAC *d*′ = 0.95 (0.73, 1.16); interaction *p* = .004; girls: NCDS *d*′ = 0.50 (0.34, 0.65), ALSPAC *d*′ = 0.99 (0.78, 1.20); interaction *p* < .001).

**Conclusions:**

Child mental health problems have become more strongly associated with negative social, educational and mental health outcomes in recent generations.

## Introduction

Child mental health problems are common, associated with distress and impairment, and can have lifelong psychosocial and health consequences (Birmaher et al., 2004; Green, McGinnity, Meltzer, Ford, & Goodman, 2005; Jokela, Ferrie, & Kivimäki, 2009; Maughan & Collishaw, 2015). It is therefore important to know whether the prevalence of child mental health problems and the outcomes for children with mental health problems have changed over time.

To date, almost all time‐trends studies of child and adolescent mental health have focused on changes in prevalence. Findings from epidemiological cross‐cohort comparisons point to substantial changes in prevalence in adolescent mental health symptoms (Collishaw, 2015; Collishaw, Maughan, Goodman, & Pickles, 2004; Collishaw, Maughan, Natarajan, & Pickles, 2010; Fleming et al., 2014; Sigfusdottir, Asgeirsdottir, Sigurdsson, & Gudjonsson, 2008; von Soest & Wichstrøm, 2014; Sweeting, Young, & West, 2009), whilst evidence on trends in younger children's mental health symptoms is equivocal (Collishaw, 2015; Matijasevich et al., 2014; Polanczyk, Willcutt, Salum, Kieling, & Rohde, 2014; Sellers, Maughan, Pickles, Thapar, & Collishaw, 2015; Sourander, Niemelä, Santalahti, Helenius, & Piha, 2008).

In contrast, very little is known about whether the consequences of child mental health problems for psychosocial functioning and longer term prognosis have changed across successive generations. To our knowledge, the only study to compare longer term outcomes across successive generations is a study of adolescents with mental health problems growing up in the 1970s or the 1980s (Collishaw et al., 2004). This study showed high and equivalent levels of pervasive adult psychosocial dysfunction for those with earlier mental health difficulties in the two cohorts. Another study considered trends in the concurrent impact of mental health symptoms in three cross‐sectional UK‐based studies in childhood (Sellers et al., 2015). Findings showed a small decline in the population prevalence of child mental health symptoms between 1999 and 2008, but an increase in concurrent functional impact on school learning, family life and social relationships for those with mental health problems.

The present study extends understanding by considering long‐term secular trends in the developmental outcomes for children with mental health problems using data from three longitudinal population cohort studies assessed over a 40‐year period (1965–2008) in the United Kingdom. The three studies – the National Child Development Study (NCDS), the Avon Longitudinal Study of Parents and Children (ALSPAC) and the Millennium Cohort Study (MCS) – provide data on mental health symptoms at age 7 years (in 1965, 1998/9 and 2008 respectively) and social, educational and mental health outcomes at ages 11 and/or 16 years.

Our main objective was to consider the extent to which risk associations changed across cohorts. To test this, we assessed interactions between cohort and child mental health in the prediction of later outcomes. Given recent evidence of increased impacts of child mental health problems on concurrent functioning (Sellers et al., 2015), we hypothesized that child mental health problems would be more strongly associated with later social, educational and mental health problems in the more recent cohorts.

## Method

The study used three longitudinal UK cohorts assessed four decades apart. In each cohort, child mental health problems were assessed at age 7 years (in 1965, 1998/9 and 2008 respectively). Information on social, educational and mental health outcomes was collected at ages 11 and/or 16 years. Prevalence rates of child mental health problems were compared for the earlier versus the two later cohorts. To examine long‐term trends in outcomes of childhood mental health difficulties, we compared social outcomes at age 11 (for the two national cohorts: NCDS, MCS) and educational and mental health outcomes for the two cohorts with age 16 follow‐up data.

### Samples

#### National Child Development Study

The National Child Development Study is a cohort of children born in one week (3^rd^ ‐ 9^th^ March) in 1958 in England, Wales and Scotland (Power & Elliott, 2005). It includes assessments at birth, ages 7, 11 and 16 years and at later adult time points (not used here). More information regarding the sample is available on the website: http://www.cls.ioe.ac.uk/. Analyses were conducted with 14,544 children (51.7% boys) with mental health data at age 7 years. Of this initial sample, 13,129 (90.3%) also had age 11 social outcome data, 14,002 (96.3%) age 16 exam data and 11,628 (80.0%) age 16 mental health data.

#### Avon Longitudinal Study of Parents and Children

ALSPAC is a longitudinal study of 14,701 children born in the Avon area (UK) with expected delivery dates between 1st April 1991 and 31st December 1992 alive at 1 year of age (Boyd et al., 2013; Fraser et al., 2013; see Appendix S1 for additional information on the ALSPAC sample). At approximately 7 years of age, an attempt was made to bolster the initial sample with eligible cases who had failed to join the study originally. This resulted in a total sample size after the age of 7 of 15,247 pregnancies, resulting in 15,458 foetuses. Of this total sample of 15,656 foetuses, 14,973 were live births and 14,899 were alive at 1 year of age. The sample is broadly representative of the UK population (Boyd et al., 2013). Please note that the study website contains details of all the data that are available through a fully searchable data dictionary and variable search tool: http://www.bris.ac.uk/alspac/researchers/our-data/. Analyses were restricted to those with available parent reports of child mental health at age 7 (*n* = 8,188/14,973; 51.3% boys). Age 16 school examination data were available for 7,484 children (84.7% of the age 7 sample) and age 16 mental health data for 4,793 children (58.5% of the age 7 sample).

#### Millennium Cohort Study

The Millennium Cohort Study is a national birth cohort of children born between 1^st^ September 2000 and 11th January 2002 in England, Wales, Scotland and Northern Ireland (Connelly & Platt, 2014). More information is available at: http://www.cls.ioe.ac.uk/. Analyses included 13,192 families who had data at age 7 years (51.4% boys). Of these families, 11,109 (84.2% of age 7) had data on social functioning at 11 years.

Ethical approval for the ALSPAC study was obtained from ALSPAC Ethics and Law Committee and the Local Research Ethics Committees. MCS was approved by the London Multi‐Centre Research Ethics Committee. NCDS received permission from Institutional Review Boards.

### Measures

#### Child mental health problems (7 years)

Child mental health problems were assessed using parent‐report screening questionnaires in all three cohorts. The Strengths and Difficulties Questionnaire (SDQ) is a well‐validated child mental health screen, and this was used in ALSPAC and MCS (Goodman, 2001). Individual items were rated on three‐point response scales (0 – ‘not true’, 1 – ‘somewhat true’, 2 – ‘certainly true’). Total problem scores (range: 0–40) reflect difficulties in four common domains of child mental ill health: emotional, conduct, hyperactivity and peer problems (each domain assessed using five items). Children were rated as having mental health problems at age 7 if they scored in the ‘abnormal range’ (≥17) of the SDQ total score. The NCDS used the precursor to the SDQ, the Rutter‐A scale (Elander & Rutter, 1996). It included 11 closely comparable items (Appendix S1 and Table S1). Despite similarities across the two measures, we considered it important to account for minor variations in item wording and response scale in undertaking cross‐cohort comparisons (Collishaw et al., 2004; Curran et al., 2008; Goodman, Iervolino, Collishaw, Pickles, & Maughan, 2007). We therefore undertook a calibration study in which data were collected from Child and Adolescent Mental Health Services and primary schools in England and Wales (*n* = 263; 53% boys; mean age = 6.48 years (*SD* = 0.72)). Parents in the calibration study completed both the SDQ and NCDS Rutter‐A scale in counter‐balanced order (see Appendix S1, for further details). There was a high correlation between Rutter‐A and SDQ total scores within the calibration sample (boys: *r* = .80; girls: *r* = .77). Data from the calibration sample were used to impute age 7 SDQ total scores for the 1958 cohort (NCDS) using available Rutter‐A scale data (total and individual item scores). To account for uncertainty in the calibration, we used 20 imputed datasets with variation in scores reflecting the probability of response in the original calibration dataset.

#### Social functioning (age 11)

Child social functioning was assessed by parents and/or children at age 11 in the two national cohorts (NCDS and MCS) with two items assessing social isolation and peer victimization (Appendix S1). These were combined (range 0–4) with higher scores reflecting greater levels of social functioning difficulties.

#### Education (age 16)

Age 15/16 school examination data were available for NCDS and ALSPAC. Academic success was defined as five or more O‐level/CSE passes (grades A–C) and five or more GCSE/NVQ passes (grades A*–C) respectively.

#### Adolescent mental health (age 16)

Adolescent mental health was assessed by parents at age 16 in NCDS and ALSPAC using the Rutter‐A scale and SDQ. Data from a separate adolescent calibration sample (Collishaw et al., 2004) were used to generate SDQ‐equivalent total problem scores for adolescents in NCDS on the basis of Rutter‐A scale measures (see Appendix S1). Total SDQ problem scores (range 0–40) were converted into *z*‐scores prior to analyses, with higher problem scores reflecting a greater number of overall symptoms.

#### Demographic variables

Parents in each cohort provided information about children's ethnic background, age 7 family status (intact vs. nonintact), age 7 housing (mortgage or home ownership vs. other), and occupation of the main earner (manual vs. nonmanual).

#### Attrition and design weights

Selective attrition in longitudinal cohort studies can affect the representativeness of retained samples (Martin et al., 2016; Taylor et al., 2018; Wadsworth et al., 2003). To address this, prospective data available for the complete age 7 samples were used to model nonresponse at follow‐up separately for each cohort and used to derive sample‐specific inverse probability weights (Appendix S2 and Tables S2‐S3).

### Analyses

Analyses were conducted separately for boys and girls. All analyses were conducted in Stata version 13 using the survey command and sample‐specific weights to account for survey design and sample attrition (Appendix S2). Analyses were conducted using the MIM command in Stata (Royston, Carlin, & White, 2009). This command combines parameter estimates from across the 20 imputed datasets. The reported results reflect both within‐dataset variation in parameter estimates (standard errors) and between‐dataset variation in parameter estimates (calibration uncertainty) and are conservative in this setting.

Analyses compared rates of children with mental health problems in each cohort (i.e. scoring in the ‘abnormal range’ of the SDQ total problem scale, ≥17, at age 7) using logistic regression, both unadjusted and adjusted for variation in demographic factors (family type, tenure, and parental occupation). Next, ordinal logistic, logistic and linear regression analyses examined associations between age 7 mental health status (above SDQ threshold or not) and age 11 social functioning difficulties (0–4), educational attainment at age 16 (5 +  exam passes vs. lower) and age 16 mental health (standardized parent total SDQ score). Tests of interactions by cohort examined changes in the strength of these associations across time.

## Results

There were marked differences in the demographic profiles of the earlier versus later cohorts (Appendix S3, Table S4). Children in the earlier cohort (NCDS, assessed at age 7 in 1965) were more likely to live in intact two‐parent families, to live in rented accommodation and to have parents in manual occupations when compared to children in the two later cohorts (ALSPAC, assessed at age 7 in 1999 and MCS, assessed at age 7 in 2008), all *p* values < .01).

### Trends in child mental health problems (age 7): 1965–2008

**Figure 1:**
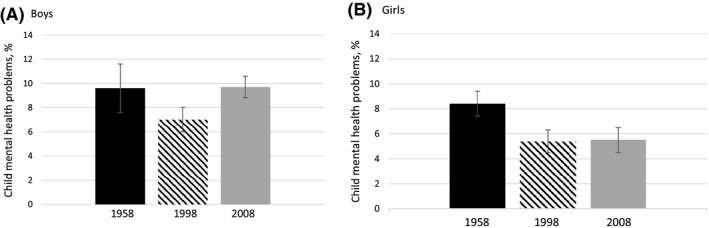
Percentage of boys (A) and girls (B) in each cohort with mental health problems at age 7 years (defined as scoring within the abnormal range, ≥17 of the Strengths and Difficulties Questionnaire). 1965 (National Child Development Study); 1998 (Avon Longitudinal Study of Parents and Children); 2008 (Millennium Cohort Study). Error bars indicate 95% confidence intervals

The prevalence of mental health problems did not increase over this period (see Figure 1). Comparing the two later cohorts with the earlier cohort showed no differences in rates of problems for boys [NCDS vs. ALSPAC: 9.6% vs. 7.0%, OR = 0.72 (95% CI 0.46, 1.13), *p* = .146; NCDS vs. MCS: 9.6% vs. 9.7%, OR = 1.01 (0.84, 1.23), *p* = .876]. Estimated rates were lower in the later cohorts for girls [NCDS vs. ALSPAC: 8.4% vs. 5.4%; OR = 0.63 (0.43, 0.92), *p* = .019; NCDS vs. MCS: 8.4% vs. 5.5%; OR = 0.78 (0.65, 0.94), *p* = .009]. When comparing the two more recent cohorts, for boys, there was a significant difference between ALSPAC and MCS (ALSPAC 7.0%, MCS 9.7%, OR = 1.43 (1.19, 1.71), *p* < .001). For girls, there was no significant difference in the proportion scoring in the abnormal range [ALSPAC 5.4%, MCS 5.5%, OR = 0.97 (0.68, 1.06), *p* = .158].

### Social functioning difficulties at age 11

Social outcomes were assessed at age 11 in the two national cohorts (NCDS in 1969 and MCS in 2011). Boys and girls with mental health problems at age 7 were more likely to report social functioning difficulties – peer victimization and social isolation – in middle childhood when compared to those without child mental health problems. This was true for both cohorts (see Table 1). Associations between child mental health and social functioning difficulties at age 11 became more marked in the more recent cohort relative to the earlier cohort for boys [cohort interaction: OR = 1.40 (1.29, 1.52), *p* < .001] and girls [cohort interaction: OR = 1.57 (1.41, 1.75), *p* < .001].

**Table 1:** Association between child mental health problems (SDQ ≥ 17 at age 7) and social functioning difficulties (age 11) in NCDS and MCS for boys and girls

	NCDS			MCS			Cohort interaction
	Child mental health problems			Child mental health problems			
	No %	Yes %	OR (95% CI)	No %	Yes %	OR (95% CI)	OR (95% CI)
Boys							
Social problems			1.95 (1.50, 2.53)**			3.77 (2.89, 4.92)**	1.40 (1.29, 1.52)**
0	20.0	14.1		29.7	12.9		
1	37.4	28.2		39.3	28.9		
2	30.8	33.7		23.7	27.9		
3	9.8	17.7		6.2	21.6		
4	1.9	6.2		1.1	8.7		
Girls							
Social problems			1.69 (1.22, 2.33)**			3.99 (3.04, 5.25)**	1.57 (1.41, 1.75)**
0	19.7	16.4		34.1	13.1		
1	41.9	32.5		40.1	28.6		
2	30.2	34.4		19.9	37.5		
3	7.2	12.1		5.0	16.1		
4	1.1	4.5		1.0	4.7		

Child mental health problems defined as Strength and Difficulties Questionnaire abnormal range scores (≥17). Social functioning difficulties score was a composite of two items assessing social isolation and peer victimization. Higher scores reflect greater levels of problems. NCDS, National Child Development Study; MCS, Millennium Cohort Study; SDQ, Strengths and Difficulties Questionnaire.

**p < .001.

### Exam attainment at age 16

Exam attainment data were available for NCDS (aged 16 in 1974) and ALSPAC (aged 16 in 2008). In both cohorts, children with mental health problems were less likely to go on to achieve at least five good exam passes than children without mental health problems [boys: NCDS: 12.9% vs. 16.6%; OR = 0.49 (0.31, 0.78), *p* = .003; ALSPAC: 44.7% vs. 72.8%; OR = 0.30 (0.22, 0.41), *p* < .001; girls: NCDS: 10.4% vs. 18.9%; OR = 0.50 (0.32, 0.76), *p* = .010; ALSPAC: 58.1% vs. 80.2%; OR = 0.34 (0.23, 0.50), *p* < .001]. For boys, a significant cohort interaction suggested that the association between child mental health and exam attainment was significantly stronger in the more recent cohort [OR = 0.42 (0.26, 0.68), *p* = .009]. The cohort interaction was not significant for girls [OR = 0.70 (0.42, 1.17), *p* = .218].

### Mental health at age 16

As shown in Table 2, age 7 mental health problems were associated with substantially poorer mental health in adolescence, in NCDS (age 16 in 1974) and in ALSPAC (age 16 in 2008). Cohort by child mental health status interactions indicated that effect sizes were significantly greater in the later cohort.

**Table 2:** Association between child mental health problems (SDQ ≥ 17 at age 7) and adolescent mental health symptom scores (standardized SDQ total scores at age 16) in NCDS and ALSPAC for boys and girls

	SDQ age 16 years		
	NCDS	ALSPAC	Cohort interaction
	*d*′ (95% CI)	*d*′ (95% CI)	
Boys	0.55 (0.38, 0.72)**	0.95 (0.73, 1.16)**	0.40 (0.12, 0.67)*
Girls	0.50 (0.34, 0.65)**	0.99 (0.78, 1.20)**	0.49 (0.23, 0.76)**

Child mental health problems defined as Strength and Difficulties Questionnaire abnormal range scores (≥17). NCDS, National Child Development Study; ALSPAC, Avon Longitudinal Study of Parents and Children; SDQ, Strengths and Difficulties Questionnaire. *d*′: Effect size.

**p < .001; *p < .05.

### Secondary analysis

#### Do cross‐cohort differences in outcomes reflect pre‐existing differences in symptom scores?

Further analyses tested whether children with mental health problems in the two later cohorts already showed greater levels of symptomatology at age 7 as this might explain cross‐cohort differences in outcomes. There was no evidence that this was the case. Table 3 shows the mean symptom scores of boys and girls with mental health problems at age 7. Symptom scores of boys with mental health problems at age 7 did not differ between NCDS and ALSPAC, nor between NCDS and MCS. For girls with child mental health problems, there was evidence that scores may be *lower* in ALSPAC compared to NCDS, but there was no significant difference between NCDS and MCS.

**Table 3:** Mean age 7 SDQ symptom scores by cohort for those defined as having mental health problems at age 7 years

	NCDS	ALSPAC	MCS	ALSPAC vs. NCDS	MCS vs. NCDS
	SDQ symptom score	SDQ symptom score	SDQ symptom score	*b* (95% CI), *p*	*b* (95% CI), *p*
	Mean (95% CI)	Mean (95% CI)	Mean (95% CI)		
Boys	21.96 (20.30, 23.61)	20.32 (19.79, 20.84)	20.80 (20.47, 21.13)	−1.64 (−3.32, 0.05), *p* = .057	−1.16 (−2.78, 0.48), *p* = .158
Girls	20.34 (19.47, 21.20)	19.30 (18.87, 19.73)	19.98 (19.64, 20.33)	−1.03 (−1.98, −0.09), *p* = .032	−.36 (−1.28, 0.56), *p* = .438

Child mental health problems defined as Strength and Difficulties Questionnaire abnormal range scores (≥17). NCDS, National Child Development Study; ALSPAC, Avon Longitudinal Study of Parents and Children; MCS, Millennium Cohort Study; SDQ, Strengths and Difficulties Questionnaire.

#### Cross‐cohort comparison of associations between child mental health and outcomes: adjusting for demographic differences

Measures of social disadvantage were more strongly associated with child mental health problems in the two recent cohorts (ALSPAC and MCS) compared with NCDS (Appendix S3, Table S5). We therefore tested the impact on our primary analyses of including social background measures as covariates. The pattern of results remained the same after adjusting for parental occupational status, housing tenure and family type (Appendix S3, Table S6).

#### Uncalibrated cross‐cohort comparisons

To examine sensitivity of the findings to the calibration, we tested cross‐cohort change in child mental health outcomes using uncalibrated child mental health scores (see Appendix S4, Table S7). We identified child mental health problems using a top 10% threshold for the Rutter‐A scale (≥11) in NCDS and the standard validated cut‐point for the SDQ (≥17). The pattern of results was the same for all outcomes, with the exception that the cohort interaction for boys’ mental health problems at age 16 was no longer significant.

## Discussion

The current study is the first to test secular change in the longitudinal outcomes of children with mental health problems. Compared to their peers, children with mental health problems in all three cohorts were considerably more likely to experience social functioning difficulties (isolation and peer victimization) later in childhood, perform more poorly academically (as reflected by formal public examination results at age 16), and experience higher rates of mental health problems in adolescence. Importantly, these associations became more pronounced over time.

In considering whether outcomes have worsened for children with mental health problems in more recent decades, it is first necessary to consider the possibility that methodological artefact – in part or in whole – accounts for the cross‐cohort differences in associations. Studies of population trends rest on the assumption that like is compared with like. The study design is the closest we have to meeting this assumption – it compared unselected population cohorts prospectively followed from early in life into adolescence using broadly comparable measures of child mental health and later outcomes. Nevertheless, differences in methodology may have affected the findings – notably changes in the specific instruments used to assess child mental health and associated longitudinal outcomes. We used data from calibration studies to mitigate this possibility. We also considered whether cross‐cohort differences in developmental outcomes might reflect pre‐existing differences in childhood symptom levels. We found no evidence that this was the case. Increased adverse outcomes observed for children with mental health problems cannot therefore be ascribed to pre‐existing differences in childhood symptom levels.

It is also possible that there have been real changes in outcomes for children with mental health problems. This is supported by the convergence of findings across diverse outcomes. If this is the case, then it would suggest that society today has become more challenging for children growing up with mental health problems. What is puzzling is that the period covered by this study has seen multiple changes in educational and social policy aimed at improving child well‐being. Progress has also been made in developing evidence‐based therapies and preventative interventions to underpin efforts to help children with mental health difficulties. In addition, many schools now have antibullying policies and programmes, parenting interventions for children with behavioural problems have become more widely available, and there is increased recognition of child psychiatric problems in clinical practice (Collishaw, 2015). It might therefore have been expected that outcomes for children with mental health difficulties would have improved for more recent generations. However, our findings suggest that negative outcomes have become more – not less – pronounced for children with mental health problems today. Understanding why such changes have occurred is an urgent priority.

First, whilst it was not possible in the current study to test specific explanations, there have been major societal changes that have likely had important impacts on the outcomes for children with mental health problems. There are substantial and widening social inequalities in children's physical and mental health (Collishaw, Furzer, Thapar, & Sellers, 2019; Gore‐Langton, Collishaw, Goodman, Pickles, & Maughan, 2011; Royal College of Paediatrics and Child Health, 2017), and recent public spending cuts have disproportionately impacted household incomes and access to support services for the most disadvantaged children in society (Stuckler et al., 2017). Second, the period of the current study has seen a remarkable shift in the educational landscape. Achieving university‐entry school exam passes was rare in the first cohort but has now become the norm. The findings here suggest that the expansion in educational opportunities has not uniformly benefited all children in society, and that those with early mental health problems are disproportionately likely to be ‘left behind’. It is also important to recognize that increased emphasis on academic success may come at a cost to young people's well‐being due to heightened academic pressure and school‐related stress (West & Sweeting, 2003). There are numerous other societal changes that might be linked with trends in child mental health outcomes including technological change (such as increased screen time and access to social media), lifestyle changes (including increased sedentary behaviour, increases in obesity and changes in sleep patterns), changes in drug and alcohol use, and earlier pubertal maturation (see e.g. Carson et al., 2016; Collishaw, 2015; Hall et al., 2016; Livingstone & Smith, 2014). Little is known about how such changes are associated with trends in child mental health prevalence, and at present there has been no research examining potential impacts on trends in outcomes for children who do have mental health problems.

## Implications for clinical practice

Analyses showed strong persistence of mental health problems between childhood and adolescence, and a worsening of outcomes in more recent cohorts. This highlights the necessity of effective screening, prevention and intervention for child mental health problems at an early age and at a population level. Population‐based evidence on service use among children with mental health problems from the UK demonstrates that only around half of children with a psychiatric disorder were in contact with public sector services of any kind about their mental health, and only a quarter were in contact with specialist mental health services (Ford, Hamilton, Goodman, & Meltzer, 2005). Given the persistence of mental health problems across childhood and adolescence, early identification and short‐term intervention in childhood may not on its own be sufficient. Continued monitoring of young people's mental health as they grow up, and long‐lasting access to relevant mental health supports are required. Finally, long‐term tracking of children's outcomes into adulthood is needed. It is well established that there is substantial continuity of mental health difficulties across the life span, but it is unknown whether this is also greater for more recent generations.

## Strengths and limitations

This study capitalized on the unique opportunity provided by the availability of three unselected population cohorts with longitudinal data about child mental health and later outcomes. There are also important limitations. First, the three studies used closely similar but not identical assessments of child and adolescent mental health. Even minor variations in question wording can influence informant response (Goodman et al., 2007). To address this, as in previous time‐trends studies (Collishaw et al., 2004), we used a calibration approach to estimate mental health scores at age 7 and age 16 for the first cohort. This analytic method is considered a conservative approach because it uses multiple imputation to appropriately take account of uncertainty in calibration estimates. Furthermore, sensitivity analyses using noncalibrated scores demonstrated a similar pattern of results. There were also differences in the wording of items assessing social functioning (peer victimization and social isolation). Here, relevant calibration data were not available and due caution is needed in interpreting the findings. In addition, different reporters completed questions regarding one indicator of social functioning (victimization was assessed using parent reports in NCDS and child reports in MCS). Whilst there may be discrepancies between parent and child reports of victimization (Matsunaga, 2009; Rønning et al., 2009), evidence suggests that both mother and child reports provide valid and reliable information on victimization and that the two are similarly associated with measures of child mental health (Shakoor et al., 2011).

Second, there were differences in sampling for the three cohorts: the 1958 and 2000/1 birth cohorts (NCDS and MCS) are national samples whilst the 1990s cohort (ALSPAC) is a regional sample (albeit broadly nationally representative in terms of sample demographics; Boyd et al., 2013). The 1958 cohort (NCDS) included children born in a single week, whilst the later cohorts sampled births across the year (potential season of birth effects seem unlikely but cannot therefore be ruled out).

A third limitation is that patterns of nonresponse differed between cohorts (with greater attrition for the more recent cohorts). There is widespread evidence that there is selective drop‐out of individuals with increased risk of mental health problems and adverse outcomes in these and other cohorts (Martin et al., 2016; Taylor et al., 2018; Wadsworth et al., 2003). To address this, we used cohort‐specific nonresponse weights.

Fourth, findings were stratified by gender, and taken together pointed to broadly consistent conclusions for boys and girls. However, as we did not have predefined hypotheses regarding gender differences in trends in outcomes, we chose not to test three‐way interactions between gender, cohort and child mental health status, and so are unable to reach firm conclusions regarding possible gender differences in trends.

Finally, it is important to consider implications of possible cross‐cohort differences in parental reporting of child mental health. It has been suggested that parents today may be more open about reporting mental health difficulties in their children. We found no evidence for this. If thresholds at which parents rated children as having mental health problems had relaxed over time (i.e. children with less severe underlying problems being more likely to meet the symptom screen threshold today) then a lower burden in terms of deleterious developmental outcomes would be expected – the opposite of what this study showed. Despite some evidence indicating a reduction in stigma associated with mental health problems in Western countries (Gilman et al., 2017), an alternative explanation is that substantial stigma remains in more recent cohorts affecting outcomes for children with mental health problems (Angermeyer & Matschinger, 2005; Chou & Mak, 1998; Crisp, Gelder, Goddard, & Meltzer, 2005; Hinshaw, 2005; Stuart, 2008). Research is needed to consider how changes in experienced stigma relate to trends in child mental health problems and outcomes.

## Conclusions

This study highlights that child mental health difficulties are common, and that they are associated with substantial functional impairments affecting children's educational progress, later social relationships and future mental health. That these deleterious developmental impacts appear to have become more marked over time is a cause for major concern. Indeed, this study is in accord with a growing body of evidence suggesting that today's children are struggling with modern societal demands (Collishaw, 2015). Researchers, practitioners and policy makers must now consider why society today has become less well adapted for promoting the healthy development of children with mental health problems, and what measures can be taken to reverse these trends.

**Key points**

Studies examining secular changes in the prevalence of mental health difficulties in children and adolescence rarely examine whether outcomes of mental health problems have changed across time.
We examined outcomes of child mental health problems in three longitudinal population cohorts of children born across a 40‐year period.
Child mental health problems have become more strongly associated with negative social, educational and mental health outcomes in recent generations.
The study highlights the importance of continued monitoring of mental health as children and young people grow up, and long‐lasting access to relevant mental health supports.
Researchers, practitioners and policy makers must now consider societal changes that have contributed to the poor outcomes for children with mental health problems today.

## Supporting information


**Appendix S1.** Measures.
**Appendix S2.** Sampling and nonresponsive weights.
**Appendix S3.** Supplementary analyses.
**Appendix S4.** Sensitivity analysis: comparison of trends in outcomes for calibrated analyses (main text) and uncalibrated analyses.
