# Supplementary material for: Cross‐cohort change in adolescent outcomes for children with mental health problems
Source: J Child Psychol Psychiatry. 2019 Apr 15;60(7):813–21. doi: 10.1111/jcpp.13029 (PMC6617990; doi:10.1111/jcpp.13029)
Supplement: Supplementary file 1 — Appendix S1. Measures. Appendix S2. Sampling and nonresponsive weights. Appendix S3. Supplementary analyses. Appendix S4. Sensitivity analysis: comparison of trends in outcomes for calibrated analyses (main text) and uncalibrated analyses. [file JCPP-60-813-s001.docx]

# Online supplement/data supplement

## Appendix S1: Measures

**Child mental health problems:** The more recent cohorts (ALSPAC, MCS) employed the Strengths and Difficulties Questionnaire (Goodman, 2004), with NCDS using the Rutter A scale (Elander & Rutter, 1996). Total Strength and Difficulties Questionnaire (SDQ) scores were calculated by adding responses to five items each about child emotional, conduct, hyperactivity, and peer problems occurring in the past six months (individual items were rated 0 ‘not true’, 1 ‘somewhat true’ or 2 ‘certainly true’). The parent Rutter A included 11 broadly comparable items each rated on a 3-point response scale (0 ‘never’, 1 ‘sometimes’ and 2 ‘frequently’).

Supplementary Table S1. Items in the SDQ and related items in the Rutter A scale as used in NCDS at age 7 and age 16

| subscale | SDQ | Abbreviated Rutter A Scale |
| --- | --- | --- |
| Emotional problems | Often complains of headaches | -- |
|  | Many worries | Worries about many things |
|  | Often unhappy, downhearted | Is miserable or tearful |
|  | Nervous or clingy in new situations | Is upset by new situation, by things happening for first time |
|  | Many fears, easily scared | -- |
| Conduct problems | Often has temper tantrums or hot tempers | Is irritable, quick to fly off the handle |
|  | Generally obedient | Is disobedient at home |
|  | Often fights with other children | Fights with other children |
|  | Often lies or cheats | -- |
|  | Steals from home, school or elsewhere | -- |
|  | -- | Destroys own or others’ belongings (e.g. tears or breaks) |
| Hyperactivity | Restless, overactive | Has difficulty in settling to anything for more than a few moments |
|  | Constantly fidgeting or squirming | Is squirmy or fidgety |
|  | Easily distracted, concentration wanders |  |
|  | Thinks things out before acting | -- |
|  | Sees tasks through to the end | -- |
| Peer Problems | Rather solitary, tends to play alone | Prefers to do things on his/her own rather than with others |
|  | Has at least one good friend |  |
|  | Generally liked by other children |  |
|  | Picked on or bullied by other children | Is bullied by other children |
|  | Gets on better with adults than with other children |  |

Evidence suggests that the Rutter A scale correlates highly with the SDQ (r = 0·88; Goodman, 2004; Muris et al., 2003; Stone et al., 2010) but it is important to account for variations in question wording and response scale when comparing the two scales (Goodman et al., 2007). We used a ‘calibration’ approach and imputed age 7 SDQ scores for the 1958 cohort (NCDS) using available Rutter-A scale data (individual item and total scale scores), drawing on separately collected data from a calibration sample where parents completed both scales. Participants in the child calibration sample were recruited from Child and Adolescent Mental Health Services and primary schools in London and Wales (n = 263; 53% boys; mean age 6·48, SD = 0·72) and were asked to complete the two questionnaires in a counterbalanced order. Calibration was undertaken separately for girls and boys. The calibration sample data were then used to estimate ordinal logistic regression models to predict total SDQ scores on the basis of Rutter A scale items and total Rutter A scale scores (boys R^2^ = .65; girls R^2^ = .60). To reflect the level of uncertainty in imputation of calibrated values and variability in the population, we used multiple imputation, imputing 20 datasets (Royston, Carlin and White, 2009).

A similar approach was taken when estimating mental health symptoms scores in the 1958 cohort (NCDS) at age 16: we again used a ‘calibration’ approach to impute age 16 total SDQ scale scores for the 1958 cohort (NCDS) using available Rutter A scale data at age 16 (individual item and total scale scores), drawing on separately collected data from an existing adolescent calibration sample where parents completed both scales. Participants for this calibration sample were recruited from secondary schools (n = 219; 57% boys; mean age = 15·2 years), and referrals to psychiatric clinics in London, UK with matched controls (n = 78 patients and 87 matched controls; 48% boys; mean age = 13·2 years; Collishaw et al., 2004). Rutter scale scores in the calibration model again explained a substantial proportion of variance at age 16 (boys R^2^ = .73; girls R^2^ = .83).

**Social relationships:** Social isolation was assessed using a parent rated question: “Child prefers to do things alone” (0 ‘never’, 1 ‘sometimes’, 2 ‘frequently’) in the 1958 cohort and “Child is rather solitary, tends to play alone.” (0 ‘not true’, 1 ‘somewhat true’, 2 ‘certainly true’) in the 2000/1 cohort. Child victimization was assessed in the 1958 cohort using the parent-reported question: “Is your child being bullied?” (0 ‘no’, 1 ‘sometimes’, 2 ‘frequently’). Child victimization was assessed in MCS using the child-reported question: ‘How often do other children hurt you or pick on you on purpose?’ 0 ‘never’, 1 ‘sometimes’ (‘every few months’ or ‘once a month’), and 2 ‘frequently’ (‘once a week’ or ‘most days’). The social functioning difficulties score combines scores across the social isolation and peer victimization items (range = 0-4), with higher scores indicating greater problems.

***Additional ALSPAC data collection information***: Pregnant women resident in Avon, UK with expected dates of delivery 1st April 1991 to 31st December 1992 were invited to take part in the study. The initial number of pregnancies enrolled is 14,541 (for these at least one questionnaire has been returned or a “Children in Focus” clinic had been attended by 19/07/99). Of these initial pregnancies, there was a total of 14,676 foetuses, resulting in 14,062 live births and 13,988 children who were alive at 1 year of age. When the oldest children were approximately 7 years of age, an attempt was made to bolster the initial sample with eligible cases who had failed to join the study originally. As a result, when considering variables collected from the age of seven onwards (and potentially abstracted from obstetric notes) there are data available for more than the 14,541 pregnancies mentioned above. The number of new pregnancies not in the initial sample (known as Phase I enrolment) that are currently represented on the built files and reflecting enrolment status at the age of 24 is 904 (452, 254 and 198 recruited during Phases II, III and IV respectively), resulting in an additional 811 children being enrolled. The total sample size for analyses using any data collected after the age of seven is therefore 15,247 pregnancies, resulting in 15,458 foetuses. Of this total sample of 15,656 foetuses, 14,973 were live births and 14,899 were alive at 1 year of age.

## Appendix S2: Sampling and non-response weights

Each cohort included information on the full cohorts studied in infancy and childhood to allow an assessment of patterns of attrition at ages 11 and 16. Characteristics of responders and non-responders in each cohort were compared in terms of data collected at birth and at age 7. Multivariable analyses demonstrated that response in the 1958 cohort (NCDS) was higher for children of married or older parents, for those whose mothers did not smoke during pregnancy, and for those with lower age 7 SDQ problem scores. In the 1991/2 cohort (ALSPAC), response was further predicted by parental housing tenure, parental education and family socioeconomic status (higher for home owners, better educated and more socially advantaged families). Non-response weights were estimated using predicted values derived from logistic regression analyses of these predictors on probability of response at each follow-up occasion separately for NCDS and ALSPAC. The top 1% of weights were trimmed to the next highest value to reduce the impact of extreme outliers. The weights adequately corrected measured biases associated with response when compared with the full samples available at birth (supplementary tables 1 and 2). The MCS design over-sampled areas with higher proportions of ethnic minorities, areas of high child poverty, and households in Scotland and Wales. Standard analytic procedures and sampling weights, developed for use with MCS (Plewis et al., 2007), accounted for this and ensured results were nationally representative. Analyses for MCS in this study were also restricted to families living in England, Scotland and Wales (in line with the NCDS cohort). Analyses of MCS included sample weights. These adjusted both for measured predictors of non-response at age 11 and corrected for sample stratification (i.e. families in different wards having an unequal probability of selection into the cohort). Further details about design, attrition weights and the representativeness of the cohort are provided elsewhere (Plewis et al., 2007).

Data Supplement Table S2. Impact of weights on cohort profile (at birth) in the 1958 cohort (NCDS)

|  | **Baseline**  **(N =18,115 )** | **Age 7 mental health**  **(N = 14,544)** | | **Age 11 social functioning**  **(N = 13,129)** | | **Age 16 mental health**  **(N = 11,628)** | | **Age 16 examinations**  **(N = 14,002)** | |
| --- | --- | --- | --- | --- | --- | --- | --- | --- | --- |
|  |  | **Unweighted** | **weighted** | **Unweighted** | **Weighted** | **Unweighted** | **Weighted** | **Unweighted** | **Weighted** |
| Birth weight (grams), mean (sd) | 3316.04  (563.02) | 3355.45  (509.16) | 3225.89  (510.01) | 3352.90  (507.17) | 3264.16  (508.02) | 3348.08  (509.16) | 3305.27  (509.72) | 3351.48  (511.99) | 3255.66  (513.69) |
| Child gender (male), % | 51.7 | 51.4 | 50.1 | 51.2 | 52.2 | 51.4 | 50.4 | 51.0 | 53.0 |
| Marital status at birth (married), % | 95.7 | 96.2 | 95.8 | 96.2 | 96.3 | 96.4 | 96.6 | 96.1 | 96.0 |
| Social class at birth (semiskilled or unskilled), % | 21.3 | 21.2 | 21.3 | 21.4 | 21.6 | 21.7 | 21.7 | 21.4 | 21.7 |
| Mother age at birth, mean (sd) | 27.43 (5.73) | 27.48 (5.68) | 27.19 (5.66) | 27.47 (5.68) | 27.39  (5.69) | 27.38  (5.63) | 27.36  (5.64) | 27.47  (5.69) | 27.41 (5.72) |
| Problems in pregnancy, % | 27.8 | 26.9 | 29.9 | 27.1 | 28.6 | 26.8 | 28.2 | 27.0 | 29.4 |
| Foetal distress, % | 9.3 | 8.6 | 10.9 | 8.7 | 10.0 | 8.7 | 9.3 | 8.9 | 9.7 |
| Maternal smoking in pregnancy, % | 33.5 | 33.0 | 34.2 | 33.2 | 34.4 | 33.1 | 33.6 | 33.3 | 34.6 |
| Mother left school at minimum leaving age, % | 74.9 | 75.1 | 74.9 | 74.5 | 75.7 | 74.9 | 74.9 | 75.0 | 75.6 |

Data Supplement Table S3. Impact of weights on cohort profile (at birth) in the 1991/2 cohort (ALSPAC).

|  | **Baseline**  **(N = 14,314)** | **Age 7**  **(N = 8,188)** | | **Age 16 SDQ total**  **(N = 4,793)** | | **Age 16 examination data**  **(N = 7,484)** | |
| --- | --- | --- | --- | --- | --- | --- | --- |
|  |  | **Unweighted** | **Weighted** | **Unweighted** | **Weighted** | **Unweighted** | **Weighted** |
| Birth weight (<2500 grams), % | 4.4 | 3.5 | 3.2 | 3.6 | 3.3 | 3.4 | 4.3 |
| child gender (male), % | 51.3 | 51.4 | 50.7 | 48.2 | 50.8 | 54.5 | 50.5 |
| Marital status at birth (unmarried, separated, divorced), % | 25.1 | 19.2 | 20.0 | 16.8 | 20.0 | 19.0 | 24.8 |
| Social class at birth (manual), % | 37.3 | 40.8 | 33.7 | 44.8 | 38.6 | 47.7 | 34.6 |
| Mother age (birth), mean (sd) | 27.96 (4.98) | 28.93 (4.61) | 27.82 (4.42) | 29.44 (4.52) | 28.71 (4.40) | 28.93 (4.65) | 27.86  (4.91) |
| Problems in pregnancy (yes), % | 25.0 | 26.4 | 26.7 | 25.8 | 26.3 | 27.4 | 25.4 |
| Smoking in pregnancy (yes), % | 30.6 | 23.3 | 27.9 | 18.2 | 21.5 | 21.9 | 30.5 |
| Maternal further education, % | 70.0 | 76.3 | 72.8 | 82.1 | 79.4 | 82.1 | 68.4 |

## Appendix S3: Supplementary analyses

Data Supplement Table S4. Demographic profile of the cohorts at age 7.

|  |  | **NCDS**  **(1958 cohort)** | | **ALSPAC**  **(1991/2 cohort)** | | **MCS**  **(2000/1 cohort)** | |
| --- | --- | --- | --- | --- | --- | --- | --- |
|  |  | Boys  % | Girls  % | Boys  % | Girls  % | Boys  % | Girls  % |
| Family status (age 7) | |  |  |  |  |  |  |
|  | Intact two-parent | 94.0 | 93.5 | 69.2 | 70.9 | 80.3 | 80.5 |
| Tenure (age 7) | |  |  |  |  |  |  |
|  | Rented | 42.2 | 42.2 | 35.9 | 36.5 | 26.8 | 26.6 |
| Occupation of main earner (birth) | | |  |  |  |  | |
|  | Manual | 69.4 | 69.5 | 30.4 | 30.2 | 33.8 | 33.1 |
| Ethnicity | |  |  |  |  |  |  |
|  | White British | 95.7 | 96.0 | 98.1 | 98.1 | 86.0 | 85.6 |

NCDS = National Child Development Study (age 7 in 1965); ALSPAC = Avon Longitudinal Study of Parents and Children (age 7 in 1998/9); MCS = Millennium Cohort Study (age 7 in 2008)

Data Supplement Table S5. Cross-cohort comparison of associations between child mental health problems (age 7) and demographic factors.

|  | **NCDS (1958 cohort)** | | | **ALSPAC (1991/2 cohort)** | | | **MCS (2000/1 cohort)** | | | **Cohort interaction, sign** |
| --- | --- | --- | --- | --- | --- | --- | --- | --- | --- | --- |
|  | **Child mental health problems** | | | **Child mental health problems** | | | **Child mental health problems** | | |  |
| **Boys** | No | Yes | OR [95% CI] | No | Yes | OR [95% CI] | No | Yes | OR [95% CI] |  |
| Family status (Intact two-parent, %) | 94.2 | 92.4 | 1.33 (.85, 2.09) | 82.0 | 68.1 | 2.13 (1.48, 3.06)** | 71.3 | 49.8 | 2.46 (2.02, 3.00)** | NCDS & ALSPAC: ns  NCDS & MCS: p <.01  ALSPAC & MCS: ns |
| Tenure (rented, %) | 57.5 | 61.3 | 0.85 (0.65, 1.12) | 19.3 | 32.9 | 0.49 (0.34, 0.69)** | 33.2 | 61.4 | 0.31 (0.26, 0.38)** | NCDS & ALSPAC: p < .05  NCDS & MCS: p < .01  ALSPAC & MCS: p < .05 |
| Occupation of main earner (manual, %) | 69.1 | 73.5 | 0.80 (0.60, 1.08) | 20.3 | 34.9 | 0.48 (0.34, 0.67)** | 33.0 | 47.8 | 0.56 (0.42, 0.76)** | NCDS & ALSPAC: p < .01  NCDS & MCS: p < .01  ALSPAC & MCS: ns |
| **Girls** |  |  |  |  |  |  |  |  |  |  |
| Family status (Intact two-parent, %) | 93.6 | 92.3 | 1.21 (0.72, 2.03) | 81.7 | 71.6 | 1.76 (1.12, 2.78)** | 72.1 | 48.9 | 2.70 (2.09, 3.49)** | NCDS & ALSPAC: ns  NCDS & MCS: p < .01  ALSPAC & MCS: ns |
| Tenure (rented, %) | 57.2 | 65.4 | 0.71 (0.52, 0.95) | 20.6 | 40.8 | 0.38 (0.26, 0.56)** | 34.8 | 65.2 | 0.29 (0.22, 0.37)** | NCDS & ALSPAC: p < .01  NCDS & MCS: p < .05  ALSPAC & MCS: ns |
| Occupation of main earner (manual, %) | 69.0 | 75.6 | 0.72 (0.52, 0.98) | 22.0 | 34.1 | 0.54 (0.36, 0.82)* | 32.5 | 49.5 | 0.49 (0.35, 0.69) | NCDS & ALSPAC: p < .05  NCDS & MCS: p < .01  ALSPAC & MCS: ns |

NCDS = National Child Development Study, ALSPAC = Avon Longitudinal Study of Parents and Children, MCS = Millennium Cohort Study. Child mental health problems defined as age 7 Strength and Difficulties Questionnaire abnormal range scores (≥17).

Supplementary Table S6: Cohort by child mental health interactions; unadjusted and adjusted for demographic factors

|  | **Child social functioning (age 11)**  **Cohort interaction**  **OR (95% CI)** | **Child exam passes (age 16)**  **Cohort interaction**  **OR (95% CI)** | **Child mental health (age 16)**  **Cohort interaction**  **b (95% CI)** |
| --- | --- | --- | --- |
| boys | NCDS vs. MCS | NCDS vs. ALSPAC | NCDS vs. ALSPAC |
| Unadjusted | OR = 1.40 (1.29, 1.52)** | OR = 0.42 (0.26. 0.68)* | b= 0.40 (0.12, 0.67)* |
| Adjusted ^a^ | OR = 1.55 (1.42, 1.68)** | OR = 0.52 (0.31, 0.83)* | b = 0.42 (0.14, 0.71)* |
| girls |  |  |  |
| Unadjusted | OR = 1.57 (1.41, 1.75)** | OR = 0.70 (0.42, 1.17) | b = 0.49 (0.23, 0.76)** |
| Adjusted ^a^ | OR = 1.78 (1.60, 1.99)** | OR = 0.76 (0.43, 1.34) | b = 0.45 (0.17, 0.73)* |

^a^ Adjusted for age 7 occupational status, housing tenure and family status, * p<.05; **p<.001

## Appendix S4: Sensitivity analysis: comparison of trends in outcomes for calibrated analyses (main text) and uncalibrated analyses

Supplementary Table S7: Cohort by child mental health interactions using calibrated SDQ (≥17) or top 10% Rutter scores (≥11) to identify child mental health problems in NCDS

|  | **Child social functioning (age 11)**  **Cohort interaction**  **OR (95% CI)** | **Child exam passes (age 16)**  **Cohort interaction**  **OR (95% CI)** | **Child mental health (age 16)**  **Cohort interaction**  **b (95% CI)** |
| --- | --- | --- | --- |
| Boys | NCDS vs. MCS | NCDS vs. ALSPAC | NCDS vs. ALSPAC |
| Calibrated | 1.40 (1.29, 1.52)** | 0.42 (0.26. 0.68)** | 0.40 (0.12, 0.67)* |
| Uncalibrated | 1.30 (1.12, 1.53)* | 0.48 (0.33, 0.70)** | 0.21 (-0.05, 0.48) |
| Girls |  |  |  |
| Calibrated | 1.57 (1.41, 1.75)** | 0.70 (0.42, 1.17) | 0.49 (0.23, 0.76)** |
| Uncalibrated | 1.47 (1.24, 1.75)** | 0.85 (0.54, 1.35) | 0.36 (0.10, 0.62)** |
